# Noninvasive Image Texture Analysis Differentiates K-ras Mutation from Pan-Wildtype NSCLC and Is Prognostic

**DOI:** 10.1371/journal.pone.0100244

**Published:** 2014-07-02

**Authors:** Glen J. Weiss, Balaji Ganeshan, Kenneth A. Miles, David H. Campbell, Philip Y. Cheung, Samuel Frank, Ronald L. Korn

**Affiliations:** 1 Cancer Treatment Centers of America, Goodyear, Arizona, United States of America; 2 The Translational Genomics Research Institute (TGen), Phoenix, Arizona, United States of America; 3 Institute of Nuclear Medicine, University College London, United Kingdom; 4 Imaging Endpoints Core Lab, Scottsdale, Arizona, United States of America; 5 Virginia G. Piper Cancer Center Clinical Trials at Scottsdale Healthcare, Scottsdale, Arizona, United States of America; Department of Biomedical Sciences and Medicine, University of Algarve, Portugal

## Abstract

**Background:**

Non-invasive characterization of a tumor's molecular features could enhance treatment management. Quantitative computed tomography (CT) based texture analysis (QTA) has been used to derive tumor heterogeneity information, and the appearance of the tumors has been shown to relate to patient outcome in non-small cell lung cancer (NSCLC) and other cancers. In this study, we examined the potential of tumoral QTA to differentiate K-ras mutant from pan-wildtype tumors and its prognostic potential using baseline pre-treatment non-contrast CT imaging in NSCLC.

**Methods:**

Tumor DNA from patients with early-stage NSCLC was analyzed on the LungCarta Panel. Cases with a K-ras mutation or pan-wildtype for 26 oncogenes and tumor suppressor genes were selected for QTA. QTA was applied to regions of interest in the primary tumor. Non-parametric Mann Whitney test assessed the ability of the QTA, clinical and patient characteristics to differentiate between K-ras mutation from pan-wildtype. A recursive decision tree was developed to determine whether the differentiation of K-ras mutant from pan-wildtype tumors could be improved by sequential application of QTA parameters. Kaplan-Meier survival analysis assessed the ability of these markers to predict survival.

**Results:**

QTA was applied to 48 cases identified, 27 had a K-ras mutation and 21 cases were pan-wildtype. Positive skewness and lower kurtosis were significantly associated with the presence of a K-ras mutation. A five node decision tree had sensitivity, specificity, and accuracy values (95% CI) of 96.3% (78.1–100), 81.0% (50.5–97.4), and 89.6% (72.9–97.0); respectively. Kurtosis was a significant predictor of OS and DFS, with a lower kurtosis value linked with poorer survival.

**Conclusions:**

Lower kurtosis and positive skewness are significantly associated with K-ras mutations. A QTA feature such as kurtosis is prognostic for OS and DFS. Non-invasive QTA can differentiate the presence of K-ras mutation from pan-wildtype NSCLC and is associated with patient survival.

## Introduction

There has been a new push for molecular characterization of tumors towards identifying a potential vulnerability for targeted therapy. Nearly all modalities to assess tumors require collection of tissue by invasive means. When a tumor sample is exhausted, a new invasive procedure would be required to analyze the molecular signature of a cancer. Recently, a “liquid biopsy” to analyze circulating tumor DNA is emerging as a potential tool for clinical application [1]. Since imaging is often used to track responsiveness to treatment and assist with clinical treatment decision-making, there is potential to evaluate tumor characteristics on imaging towards non-invasive characterization of the tumor's molecular genotype. Recently, researchers have investigated tumor heterogeneity on imaging to assess how grainy or coarse a tumor seems to be in the search for oncologic prognostic markers and mechanisms. Quantitative computed tomography (CT) based texture analysis (QTA) has been used to derive tumor heterogeneity information, and the appearance of the tumors has been shown to relate to patient outcome in esophageal, colorectal, lung and head and neck cancer and treatment response in metastatic renal cell cancer [2]. Furthermore histological assessment has demonstrated an association between QTA and hypoxia and angiogenesis in lung cancer and very recently QTA in combination with CT blood-flow and PET glucose-uptake identified an imaging signature for K-ras mutation status in colorectal cancer [3].

Worldwide lung cancer is the leading cause of cancer-related mortality, responsible for nearly 1.4 million deaths annually [4]. Approximately 85% of lung cancers are non-small cell lung cancer (NSCLC) and about 67% present with advanced disease. With further refinement of treatment decisions utilizing molecular analysis, rapid identification of molecular targets associated with resistance or responsiveness to molecularly based therapies in a non-invasive manner for advanced NSCLC can improve treatment efficiencies by providing go-no go decisions faster than or complementary with traditional and/or evolving laboratory assay techniques. In this study, we examined the potential of tumoral QTA to differentiate K-ras mutant from pan-wildtype tumors and its prognostic potential using non-contrast CT imaging in NSCLC.

## Materials and Methods

### Ethics Statement

After obtaining approval of the Scottsdale Healthcare IRB under Exemption 4 of Title 45 Code of Federal Regulations (CFR) concerning retrospective study of existing data, qualifying lung tumor tissues were collected. Under exemption 4, patient consent is not required and was not obtained for this study, as Title 45 CFR Part 46 does not apply. Specimens were de-identified and the clinical information associated with these specimens is not individually identifiable such that subjects cannot be identified either directly or indirectly through identifiers linked to the subjects.

### Tumor Specimens and Clinical Annotation

Formalin-fixed, paraffin-embedded (FFPE) lung tumor tissues were obtained with approval of the local IRB from patients with early-stage NSCLC (stage I and II according the AJCC 7^th^ edition), diagnosed between 2001 and 2007 and receiving follow-up care at Scottsdale Healthcare, Scottsdale, AZ as previously described [5]. Tumor DNA was extracted and analyzed on the LungCarta Panel (Sequenom, Inc.) consisting of 24 wells containing a total of 249 multiplexed assays that interrogate 213 somatic mutations in 26 oncogenes and/or tumor suppressor genes. **Table 1** lists the genes and loci analyzed.

**Table 1:** List of Genes Assessed.

Gene Name
AKT1
ALK
BRAF
DDR2
EGFR
EPHA3
EPHA5
ERBB2
FGFR4
JAK2
KRAS
MAP2K1
STK11
MET
NOTCH1
NRAS
NRF2
NTRK1
NTRK2
NTRK3
PIK3CA
PTCH1
PTEN
PTPN11
PTPRD
TP53

### CT acquisition

Cases with a K-ras mutation or pan-wildtype for all 26 oncogenes and tumor suppressor genes were selected for imaging analysis. Baseline pre-treatment non-contrast CT images that were performed as part of standard of care were retrieved for QTA with local IRB approval. In general, CT acquisition parameters were obtained by spiral technique at 140 kVp, 40–80 mAS, 3–5 mm slice thickness using soft tissue and lung reconstruction algorithms per manufacturer's specification.

### Quantitative Texture Analysis - QTA

QTA was applied to regions of interest in the primary tumor and comprised an image filtration-histogram technique previously reported in detail [6], [7]; where the filtration technique using a Laplacian of Gaussian band-pass filter enhanced and extracted features of different sizes based on the spatial scale filter (SSF) value varying from fine-texture (SSF2, 2 mm in radius), medium-texture (SSF3, 3 mm in radius), and coarse-texture (SSF4, 4 mm in radius) followed by quantification of the filtered texture maps using histogram parameters – mean (a measure of average brightness), kurtosis (K, a measure of peakedness and tailedness), skewness (S, a measure of asymmetry of the histogram) and standard-deviation (SD, a measure of variation or dispersion that exists from the mean) [8]. These histogram parameters were also quantified from the conventional CT image without filtration (i.e. SSF0). A recent article [9] further described what these texture parameters mean in terms of image features and how it relates to visually perceptible heterogeneity in terms of features/objects of different sizes, density and concentration.

### Statistical Analysis

#### Differentiation between K-ras mutation and pan-wildtype tumors

The non-parametric Mann Whitney test was used to assess the ability of the QTA, clinical, and patient characteristics to differentiate between K-ras mutation from pan-wildtype. A recursive decision tree was developed to determine whether the differentiation of K-ras mutant from pan-wildtype tumors could be improved by sequential application of QTA parameters. The QTA parameter that most accurately identified K-ras mutants in the entire patient cohort was used as the first node, thereby generating two branches. The abilities of additional QTA parameters to identify K-ras mutants where then separately tested for each branch, adding further nodes to the tree. The diagnostic threshold values for the QTA parameters used at each node were optimized using Receiver Operator Characteristic (ROC) analysis to maximize diagnostic accuracy. QTA parameters were applied in order of overall accuracy of detection of the K-ras mutation. If two modalities had the same accuracy, the modality with the highest sensitivity was applied first. No further node was added to a branch if the mis-classification rate in that branch was less than 15%. The final decision tree was applied to the whole cohort to determine the overall sensitivity, specificity, and accuracy.

A Monte Carlo analysis using 1,000 iterations of the decision tree was used to assess the impact of uncertainty in the sensitivity and specificity for each QTA parameter (expressed as their 95% confidence intervals [CI]) on the overall diagnostic performance of the decision tree. For each iteration, the sensitivity and specificity for each decision node were randomly selected from a normal distribution with +/−2 standard deviations corresponding to the 95% CI. The 2.5 and 97.5 percentiles of the resulting 1,000 values for overall sensitivity, specificity, and accuracy were used to characterize the CI for the diagnostic performance of the decision tree. The z-statistic was used to compare the misclassification rate of node 1 used alone to that for the whole decision tree.

#### Survival Analysis

Univariate Kaplan-Meier survival (KM) analysis assessed the relationship between the above markers (including K-ras mutation) and patient overall survival (OS) and disease-free survival (DFS). An iterative procedure was applied to identify the optimal cut off for each marker that provides the best separation of the population into ‘good’ and ‘poor’ prognosis groups (indicated by the best *p*-value from log-rank test). Due to small numbers, we censored reporting results for significant features yielding less than 10 patients per group for comparison to avoid overstating significant results. Additionally, we focused on analysis for SSF0 through 4, because of small numbers of remaining cases having QTA measures for SSF 5 mm in radius and above.

Multivariate Cox regression was used to determine which significant univariate parameters along with their interactions were independent predictors of survival, along with the hazard ratio (HR) and the CI. The ability of the significant markers to identify a sub-group of patients who have good survival from those with poor survival was further tested by comparing the prognostic performance of the significant markers in patients with K-ras mutation separately from patients with pan-wildtype. A two-tailed *P* value of less than 0.05 was considered to indicate a significant difference. All statistical analyses were performed using IBM SPSS Statistics for Windows version 19.0 (IBM Corp. Armonk, NY).

## Results

Forty-eight cases were identified and QTA was applied to pre-treatment non-contrast CT. The median age was 70.4 years (range 45.1–85.1), 29 were men, and 46 had a smoking history. There were 33 adenocarcinomas, 10 squamous cell carcinomas, and five other NSCLC. Stages IA, IB, IIA, and IIB were 16, 21, 6, and 5; respectively. At least 33 patients did not receive adjuvant chemotherapy. There were 27 cases with K-ras mutation and 21 cases that were pan-wildtype. As of the last follow-up, 18 had confirmed disease relapse, including five with brain metastasis, and 22 were deceased. The median disease-free survival (DFS) was 39.7 months and median overall survival (OS) was 45.0 months (**Table 2**). Among the clinical factors including: tumor size, age, gender, stage, histology, smoking history, receipt of adjuvant chemotherapy, DFS, OS, disease relapse, development of metastasis, development of brain metastasis, and survival status; only age was significantly different between cases with a K-ras mutation and those that were pan-wildtype (p = 0.048)(**Table S1**). Although adenocarcinoma was slightly more prevalent amongst cases with a K-ras mutation (74.1% versus 61.9%), this difference was not statistically significant.

**Table 2:** Dataset Clinical features.

Case	Age (Yrs)	Gender (M/F)	Initial Stage	Histology (Adeno, Squamous, Other)	Smoker? (Yes, No, Unknown)	Adjuvant Therapy (No, Yes, specific if known, Unknown)	Relapse (Yes, No, Unknown)	Brain Metastasis (Yes, No)	Vital Status (Alive, Deceased)	DFS (Mos)	OS (Mos)
K-ras1	65.2	M	IA	A	Y	N	N	N	A	61.57	61.57
K-ras2	66.2	M	IA	S	Y	N	N	N	D	27.30	27.30
K-ras3	70.6	M	IA	A	Y	N	Y	Y	D	17.90	59.60
K-ras4	65.0	F	IA	A	Y	N	N	N	A	39.73	39.73
K-ras5	71.5	M	IA	A	Y	N	N	N	A	47.27	47.27
K-ras6	68.9	F	IA	S	Y	N	N	N	A	66.70	66.70
K-ras7	53.8	F	IB	A	Y	Y	Y	Y	A	9.60	43.20
K-ras8	76.2	M	IB	A	Y	N	Y	N	D	30.13	30.73
K-ras9	62.2	F	IB	A	Y	N	N	N	A	87.03	87.03
K-ras10	64.0	F	IB	O	Y	N	N	N	D	51.17	51.17
K-ras11	45.1	F	IIB	O	Y	U	N	N	D	93.17	93.17
K-ras12	74.2	F	IB	A	Y	N	Y	N	D	12.50	19.83
K-ras13	79.7	M	IB	A	Y	N	Y	N	D	31.63	40.40
K-ras14	61.5	M	IA	A	Y	N	N	N	A	101.00	101.00
K-ras15	61.2	M	IB	O	Y	Y	N	N	A	78.10	78.10
K-ras16	69.9	F	IB	A	Y	N	Y	Y	D	34.37	89.43
K-ras17	65.6	F	IB	A	Y	N	N	N	A	79.13	79.13
K-ras18	71.3	M	IB	A	Y	U	N	N	A	73.73	73.73
K-ras19	49.7	F	IB	A	Y	U	N	N	A	66.13	66.13
K-ras20	76.6	M	IB	A	Y	U	U	U	D	U	12.63
K-ras21	72.6	F	IIA	A	Y	Y, CIS/VIN	Y	N	A	6.97	59.13
K-ras22	82.0	M	IIA	A	Y	N	Y	N	D	8.20	15.97
K-ras23	67.3	M	IA	A	Y	U	U	N	D	U	80.87
K-ras24	53.9	M	IB	A	Y	N	N	N	A	108.43	108.43
K-ras25	63.6	M	IB	S	Y	N	N	N	A	129.57	129.57
K-ras26	72.2	M	IIB	S	Y	N	Y	N	D	7.47	12.17
K-ras27	74.9	M	IIA	A	Y	Y	Y	Y	D	12.37	37.83
Pan-wildtype1	59.8	F	IA	A	Y	N	N	N	A	59.00	59.00
Pan-wildtype2	85.1	M	IA	A	N	N	Y	N	D	51.50	63.10
Pan-wildtype3	82.9	M	IA	S	Y	N	Y	N	A	58.00	96.73
Pan-wildtype4	82.5	F	IA	A	Y	N	N	N	A	44.50	44.50
Pan-wildtype5	82.2	F	IB	A	Y	N	N	N	A	2.63	2.63
Pan-wildtype6	50.1	F	IA	A	U	N	Y	Y	A	55.43	79.07
Pan-wildtype7	76.2	M	IA	S	Y	N	N	N	A	28.27	56.50
Pan-wildtype8	71.1	F	IB	A	Y	N	N	N	A	44.97	44.97
Pan-wildtype9	65.5	M	IA	S	Y	N	N	N	D	29.13	29.13
Pan-wildtype10	68.3	M	IB	S	Y	N	N	N	A	77.13	77.13
Pan-wildtype11	77.1	F	IB	A	Y	N	Y	N	D	10.03	25.27
Pan-wildtype12	46.0	F	IB	A	Y	U	N	N	A	76.47	76.47
Pan-wildtype13	72.3	M	IIA	S	Y	N	N	N	A	96.33	96.33
Pan-wildtype14	72.8	M	IIA	A	Y	Y, CIS/VIN	Y	N	D	22.80	29.83
Pan-wildtype15	66.1	F	IIB	A	Y	U	N	N	A	77.43	77.43
Pan-wildtype16	81.1	M	IIA	A	Y	U	Y	N	D	61.20	61.63
Pan-wildtype17	78.1	M	IA	O	Y	N	N	N	D	23.03	23.03
Pan-wildtype18	74.9	M	IB	A	Y	N	Y	N	D	6.73	20.90
Pan-wildtype19	70.2	M	IB	A	Y	N	U	U	D	U	71.07
Pan-wildtype20	73.3	M	IIB	O	Y	Y, CIS/ETOP	Y	N	D	23.43	27.73
Pan-wildtype21	65.0	M	IIB	S	Y	U	N	N	A	50.00	50.00

### QTA differentiating K-ras mutation from pan-wild type

**Figure 1:**
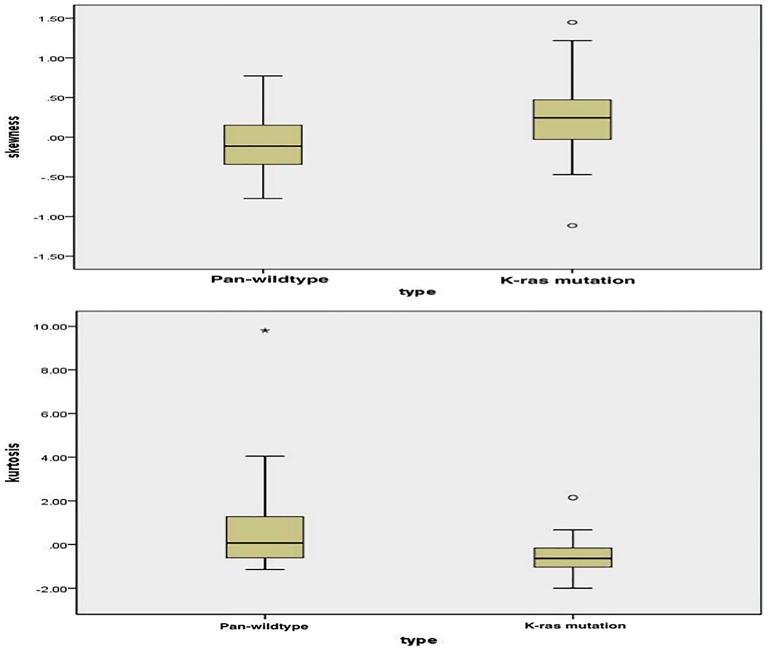
Box plot of pan-wildtype vs. K-ras mutation. **A**) **(Top Panel):** Positive skewness with fine-texture significantly differentiates K-ras mutation from pan-wildtype (p = 0.031). **B**) **(Bottom Panel):** Lower kurtosis with coarse-texture significantly differentiates K-ras mutation from pan-wildtype (p = 0.009).

**Figure 2:**
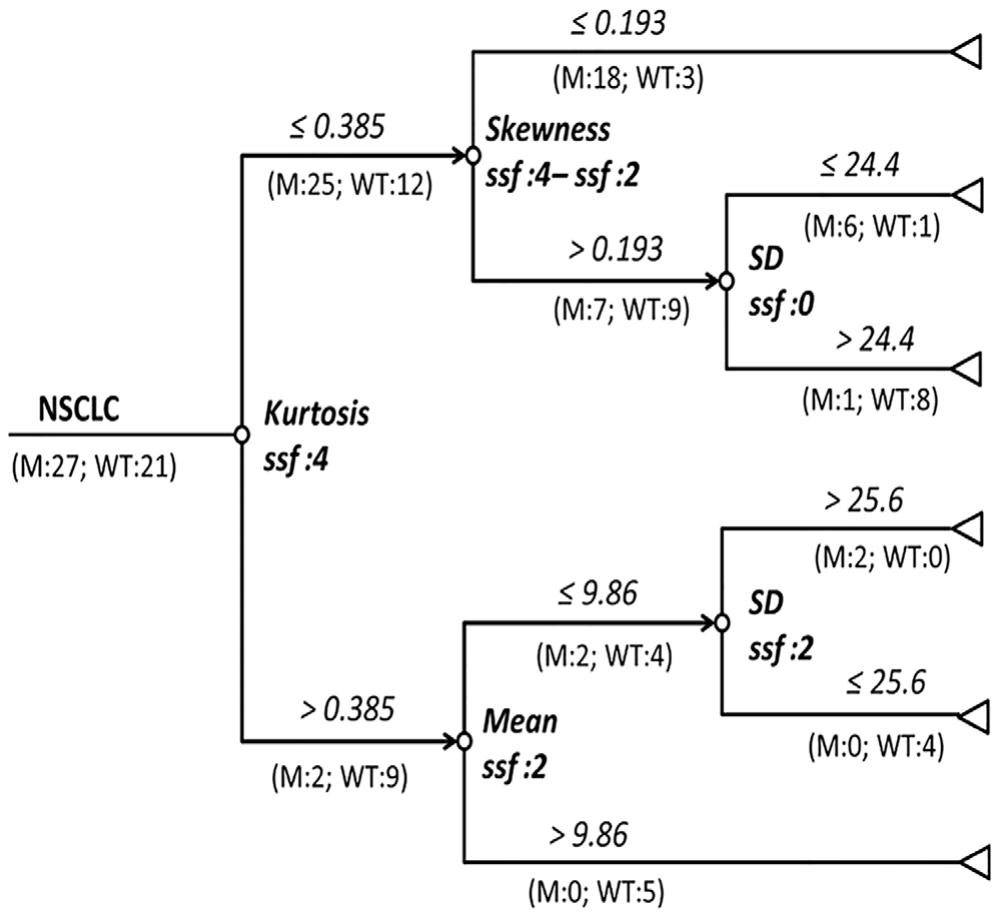
Decision-tree for identification of KRAS mutations using QTA parameters. M-Numbers of mutants; WT-number of pan-wildtype; SD-standard deviation; ssf-spatial scale factor.

From the figures (**Figure 1A–B**, representative figure depicting skewness and kurtosis displayed in **Figure S1**) positive skewness at fine-texture and lower kurtosis at coarse-texture were able to significantly differentiate K-ras mutation from pan-wildtype (p = 0.031 and p = 0.009, respectively). The final decision tree comprised five nodes (**Figure 2**). The first node of the decision tree used the QTA parameter with the highest diagnostic accuracy i.e. kurtosis at coarse-texture with a threshold value of 0.385 giving sensitivity, specificity, and accuracy values (95% CI) of 92.6% (75.7–99.1%), 42.9% (21.8–66.0%), and 70.8% (55.7–82.3%); respectively. Difference is skewness at coarse and fine-textures and mean at fine-texture were assessed second and finally SD without filtration and at fine-texture were assessed last. Thus, the final decision tree comprised 5 decision nodes and 6 terminal nodes (of which 3 identified K-ras mutation and the remaining 3 identified pan-wild type status) (**Figure 2**). From the Monte Carlo analysis corresponding values for the whole decision tree were 96.3% (95% CI 78.1–100), 81.0% (95% CI 50.5–97.4), and 89.6% (95% CI 72.9–97.0); respectively. The misclassification rate for the decision tree was significantly lower as compared to node 1 applied alone (z = 2.31, p = 0.021).

### Overall survival

**Figure 3:**
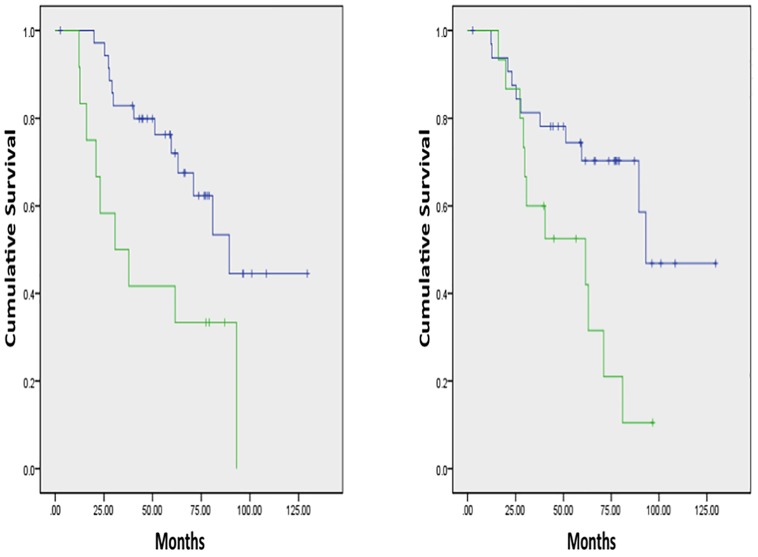
Overall survival curves based on QTA. **A**) **(Left Panel):** Without filtration, higher skewness (green line, median OS 30.7 months) vs. lower skewness (blue line, median OS 89.4 months) was associated with inferior OS (p = 0.012). **B**) **(Right Panel):** Without filtration, lower kurtosis (green line, median OS 61.6 months) vs. higher kurtosis (blue line, median OS 93.2 months) was associated with inferior OS (p = 0.012).

**Figure 4:**
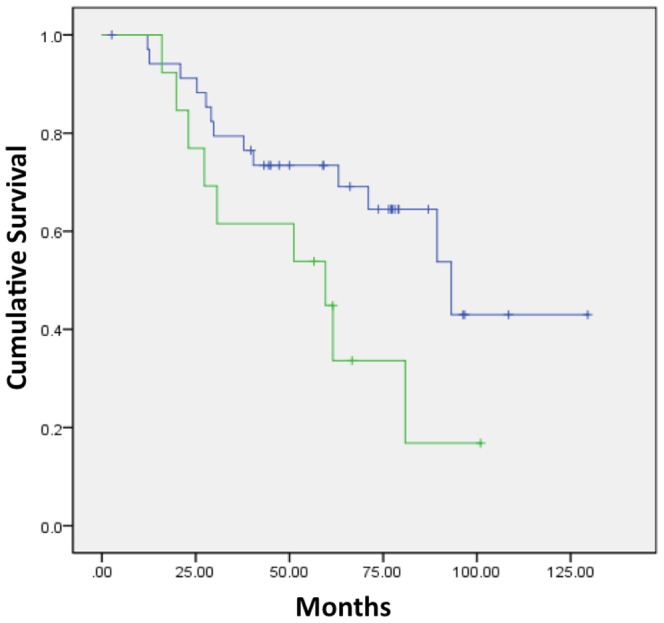
Disease free survival curves based on QTA. Lower kurtosis with coarse-texture (green line, median DFS 59.6 months) had inferior OS vs. higher kurtosis (blue line, median DFS 93.2 months)(p = 0.048).

There were no significant differences in OS between NSCLC patients possessing tumors with K-ras mutation and pan-wildtype, in line with other published data in early-stage NSCLC [10] (**Figure S2**). Without filtration, several factors were significantly associated with OS including SD, skewness, and kurtosis. It was not surprising to observe that significantly inferior OS for those patients developing disease relapse and/or metastasis and older age patients. Higher skewness without filtration (n = 12) vs. lower skewness without filtration (n = 36) was associated with inferior OS (p = 0.012), while lower kurtosis without filtration (n = 15) vs. higher kurtosis without filtration (n = 33) was associated with inferior OS (p = 0.012)(**Figure 3A–B**; respectively). Higher SD without filtration or with fine-texture was significantly associated with shorter OS (p = 0.028 and p = 0.046; respectively)(data not shown). Lower kurtosis with coarse-texture (n = 13) had inferior OS vs. higher kurtosis (n = 35)(p = 0.048)(**Figure 4**). Cox regression analysis indicated kurtosis with coarse-texture and age were independent predictors of OS (kurtosis with coarse-texture: HR, 2.547; 95% CI; 1.001–6.479; p = 0.050; age: HR, 6.022; 95% CI; 2.383–15.216; p<0.001).

### Disease-free survival

There were no significant differences in DFS between NSCLC patients possessing tumors with K-ras mutation and pan-wildtype (**Figure S3**). Without filtration, several factors were significantly associated with DFS including mean and kurtosis. Older age patients also had significantly inferior DFS. Lower mean was significantly associated with shorter DFS (p = 0.009)(**Figure S4**). Lower kurtosis (n = 13) vs. higher kurtosis without filtration (n = 32) was associated with shorter DFS (p = 0.049)(data not shown). Cox regression analysis indicated mean without filtration and age were independent predictors of DFS (mean without filtration: HR, 3.614; 95% CI; 1.325–9.855; p = 0.012 and age: HR, 9.621; 95% CI; 3.261–28.385; p<0.001).

Additionally, we looked for the prognostic effect of QTA on OS and DFS separately for patients with K-ras mutant and pan-wildtype NSCLC. Exploring K-ras mutant and pan-wildtype cases separately without filtration, several factors were significantly associated with OS including SD, kurtosis, skewness, and mean. With fine-texture, in patients with a K-ras mutant tumor, higher SD was significantly associated with shorter OS (p = 0.038), while there were no significant differences in OS with SD in patients with pan-wildtype tumors (**Figure S5A–B**; respectively). With coarse-texture, in patients with a K-ras mutant tumor, lower kurtosis was significantly associated with shorter OS (p = 0.044), while there were no significant differences in OS with kurtosis in patients with pan-wildtype tumors (**Figure S5C–D**; respectively). Without filtration in patients with a K-ras mutant tumor, lower mean was significantly associated with shorter DFS (p = 0.015)(**Figure S6A**). Without filtration in patients with a pan-wildtype tumor, there was no significant difference in DFS for mean (**Figure S6B**).

## Discussion

In this study, we sought to apply QTA analysis to molecularly defined NSCLC tumors to determine if noninvasively we could discriminate K-ras mutant from pan-wildtype cases and also determine if QTA could be used as a prognostic tool in early-stage NSCLC. We found that positive skewness with fine-texture (which may reflect bright highlighted features surrounded by darker background or lower density areas) and lower kurtosis with coarse-texture (which may reflect increased number of highlighted features and intensity variation in highlighted features) are significantly associated with K-ras mutations. These features may suggest more focal fibrosis and these may be due to the hostile microenvironment within a K-ras driven tumor [11], [12]. Fibrosis has previously been associated with inferior outcomes for both squamous cell carcinoma and adenocarcinoma of the lung [13], [14]. The misclassification rate for identification of KRAS mutations can be reduced by deploying a decision that serially combines multiple QTA parameters. The decision tree developed using our dataset has an accuracy of 89.6% in differentiating K-ras mutant from pan-wildtype tumors.

The outcomes in this dataset are in accordance with published poor clinical prognostic features, primarily the presence/occurrence of disease-relapse and metastasis in patients originally diagnosed with early-stage NSCLC [15]. There were too few cases in this dataset receiving adjuvant chemotherapy, so meaningful interpretation on how that affected outcome is not feasible. From data reported in **Table S1**, K-ras mutant and pan-wildtype did not have a significantly different number of patients receiving adjuvant chemotherapy.

With QTA, positive skewness without filtration, lower SD without filtration or with fine-texture, and lower kurtosis with coarse-texture are prognostic for shorter OS. It is encouraging that the same QTA features and directional orientation that differentiate K-ras from pan-wildtype (skewness and kurtosis) are prognostic for OS. The main difference is that less stringent filtration settings were required to show demonstrate OS differences. Interestingly, patients with K-ras mutant tumors with higher kurtosis had no significant differences in OS from patients with pan-wildtype tumors. This finding may reflect phenotypic variability amongst K-ras mutations associated with differences in tumor aggression. Phenotypic variations with variable treatment responsiveness have been observed within K-ras mutations related to different amino acid substitutions of the mutation [16]. Although in this dataset, an argument for differential response to treatment is likely irrelevant because during the years 2001–2007, few patients were even eligible for adjuvant systemic therapy.

For DFS as with OS, older age is associated with inferior outcome. By QTA, lower mean and lower kurtosis without filtration are prognostic for shorter DFS. Interestingly, when analyzing QTA in K-ras mutant and pan-wildtype cases separately, there was further differentiation for OS amongst only the K-ras mutant cases. QTA features included SD with fine texture and kurtosis with coarse-texture. In K-ras mutant cases, consistent with the entire dataset, higher SD was associated with shorter OS.

The ability to rapidly and noninvasively characterize NSCLC tumors would be a great asset to clinical oncologists. This type of endeavor would require coordination between radiologists, pathologists, and oncologists to develop the workflow to confirm established biomarkers for NSCLC with the flexibility to be able to add newly discovered and clinically validated biomarkers [17]. QTA applied to molecularly defined NSCLC cases may have broader application to Precision Medicine by offering a noninvasive modality that could help identify appropriate or inappropriate molecularly defined targeted therapy, particularly since QTA can utilize digital images already acquired during standard of care treatment [18].

## Conclusions

Non-invasive QTA can differentiate the presence of K-ras mutation from pan-wildtype NSCLC with high sensitivity, specificity, and accuracy, as well as, associate with patient survival. Validation of these findings, applying the same thresholds and criteria, to independent NSCLC sample sets is required as the next step.

## Supporting Information

Figure S1 **Representative QTA images for kurtosis and skewness.** K-ras mutant NSCLC with lower kurtosis **(top left panel)** and more positive skewness differentiates from a pan-wildtype NSCLC **(bottom left panel). Right panel:** depicts a histogram plot of K-ras vs. pan-wildtype NSCLC
(TIF)

Figure S2 **Overall survival curves based on mutation status.** Without filtration, there were no significant differences in OS between NSCLC patients possessing tumors with K-ras mutation (green line, median OS 89.4 months) and pan-wildtype (blue line, median OS 71.1 months)(p = 0.96).
(TIF)

Figure S3 **Disease free survival curves based on mutation status.** Without filtration, there were no significant differences in DFS between NSCLC patients possessing tumors with K-ras mutation (green line, median DFS not reached) and pan-wildtype (blue line, median DFS 61.2 months)(p = 0.93).
(TIF)

Figure S4 **Disease free survival curves based on QTA.** Without filtration, lower mean (green line, median DFS 55.4 months vs. blue line, median DFS not reached) was significantly associated with shorter DFS (p = 0.009).
(TIF)

Figure S5 **Overall survival curves based on QTA within K-ras mutant and pan-wildtype cases.** With fine-texture, in patients with a K-ras mutant tumor, higher SD (blue line, median OS 59.6 months vs. green line, median OS not reached) was significantly associated with shorter OS (p = 0.038)(**A, top left panel**), while there were no significant differences in OS with SD in patients with pan-wildtype tumors (higher SD, blue line, median OS 71.1 months vs. lower SD, green line, median OS not reached)(**B, top right panel)**. With coarse-texture, in patients with a K-ras mutant tumor, higher kurtosis (blue line, median OS 51.2 months vs. green line, median OS 93.2 months) was significantly associated with shorter OS (p = 0.044)(**C, bottom left panel**), while there were no significant differences in OS with kurtosis in patients with pan-wildtype tumors (higher kurtosis, blue line, median OS 61.6 months vs. lower kurtosis, green line, median OS not reached)(**D, bottom right panel**).
(TIF)

Figure S6 **Disease free survival curves based on QTA within K-ras mutant and pan-wildtype cases.** Without filtration in patients with a K-ras mutant tumor, lower mean (green line, median DFS 17.9 months vs. blue line, median DFS not reached) was significantly associated with shorter DFS (p = 0.015)(**A, left panel**). Without filtration in patients with a pan-wildtype tumor, there was no significant difference in DFS (green line, median DFS 61.2 months vs. blue line, median DFS 58.0 months)(**B, right panel**).
(TIF)

Table S1 **Clinical characteristics comparing pan-wildtype and K-ras mutant.**
(DOCX)
