# Supplementary figures and images for: Noninvasive Image Texture Analysis Differentiates K-ras Mutation from Pan-Wildtype NSCLC and Is Prognostic
Source: PLoS One. 2014 Jul 2;9(7):e100244. doi: 10.1371/journal.pone.0100244 (PMC4079229; doi:10.1371/journal.pone.0100244)

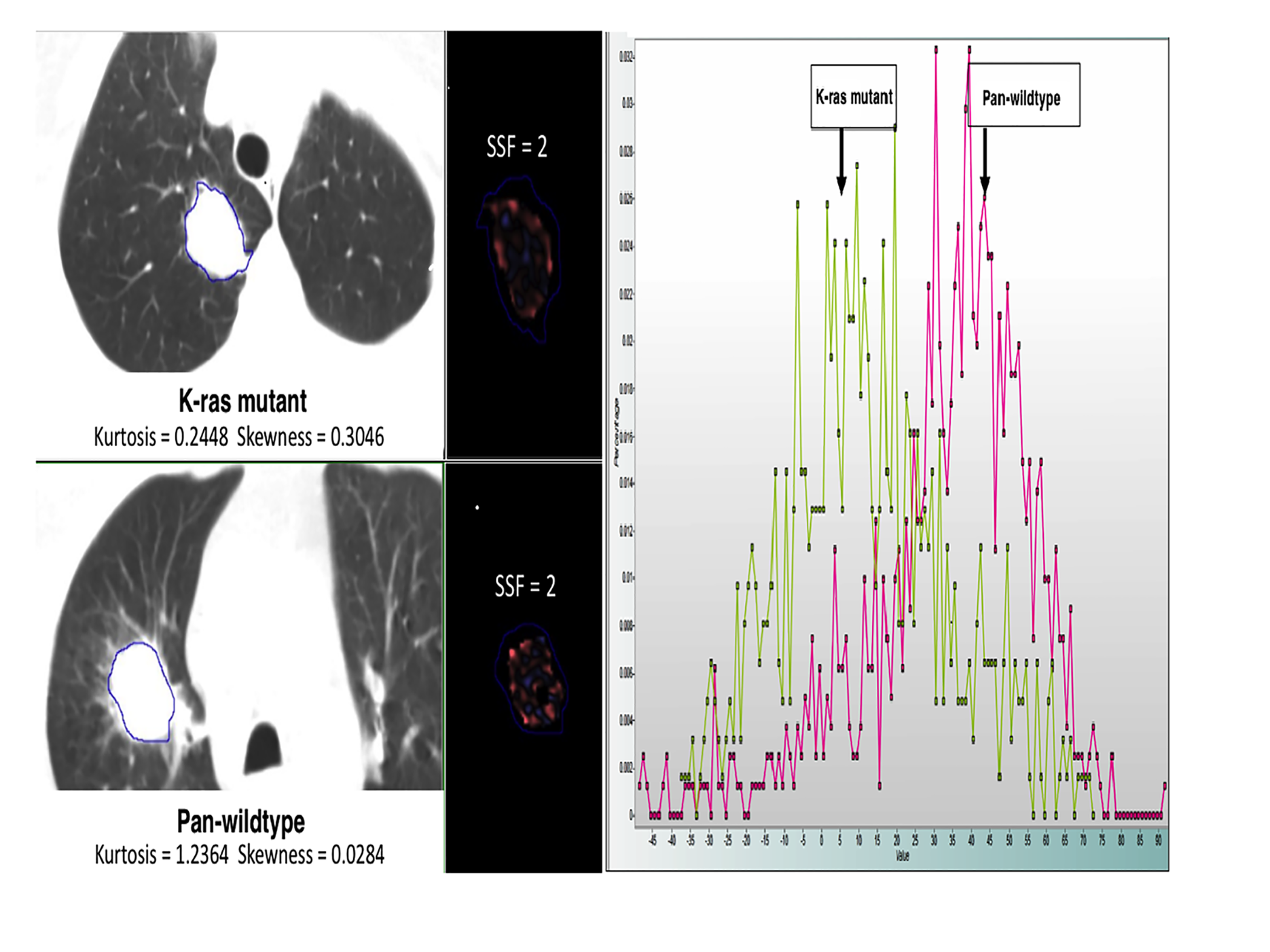

Supplement: Figure S1 — Representative QTA images for kurtosis and skewness. K-ras mutant NSCLC with lower kurtosis (top left panel) and more positive skewness differentiates from a pan-wildtype NSCLC (bottom left panel). Right panel: depicts a histogram plot of K-ras vs. pan-wildtype NSCLC (TIF) [file pone.0100244.s001.tif]

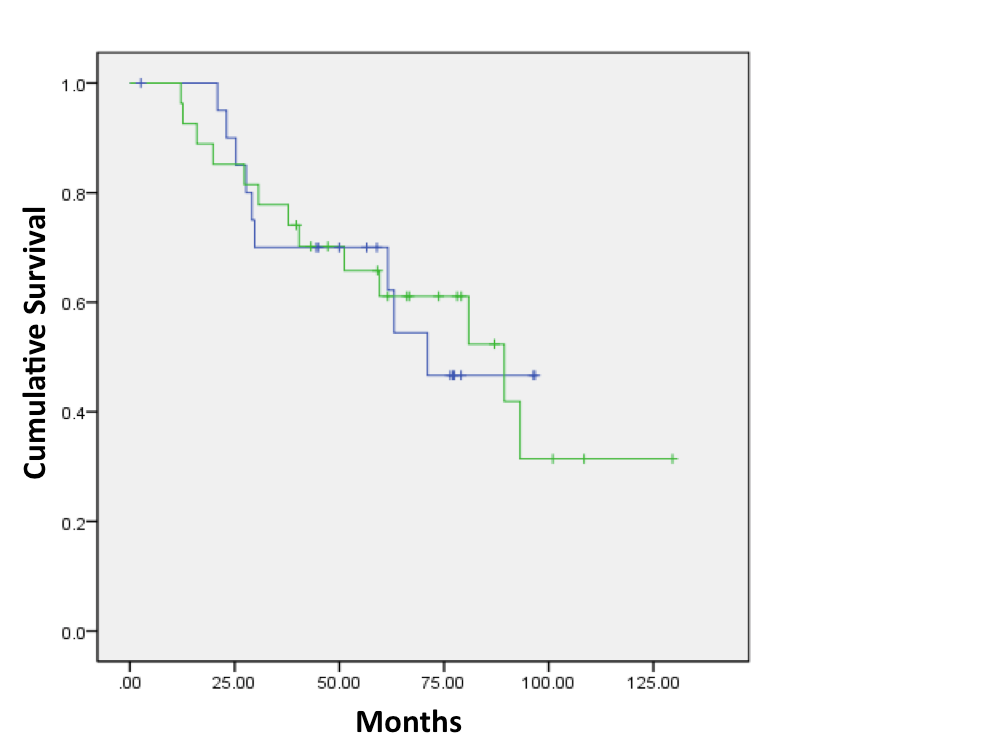

Supplement: Figure S2 — Overall survival curves based on mutation status. Without filtration, there were no significant differences in OS between NSCLC patients possessing tumors with K-ras mutation (green line, median OS 89.4 months) and pan-wildtype (blue line, median OS 71.1 months)(p = 0.96). (TIF) [file pone.0100244.s002.tif]

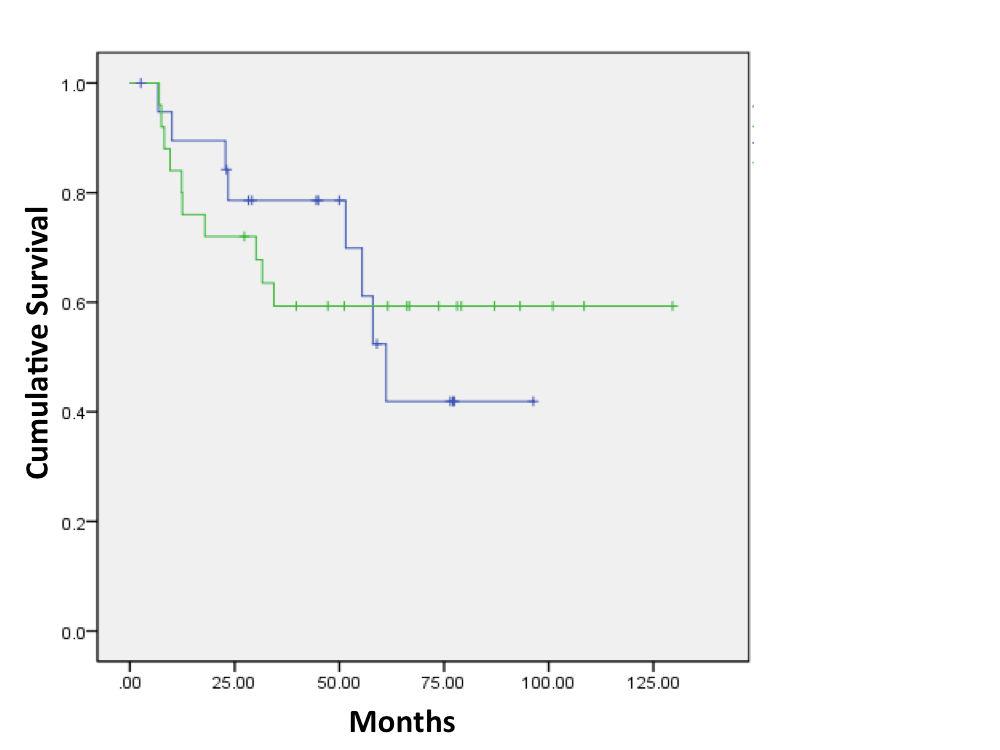

Supplement: Figure S3 — Disease free survival curves based on mutation status. Without filtration, there were no significant differences in DFS between NSCLC patients possessing tumors with K-ras mutation (green line, median DFS not reached) and pan-wildtype (blue line, median DFS 61.2 months)(p = 0.93). (TIF) [file pone.0100244.s003.tif]

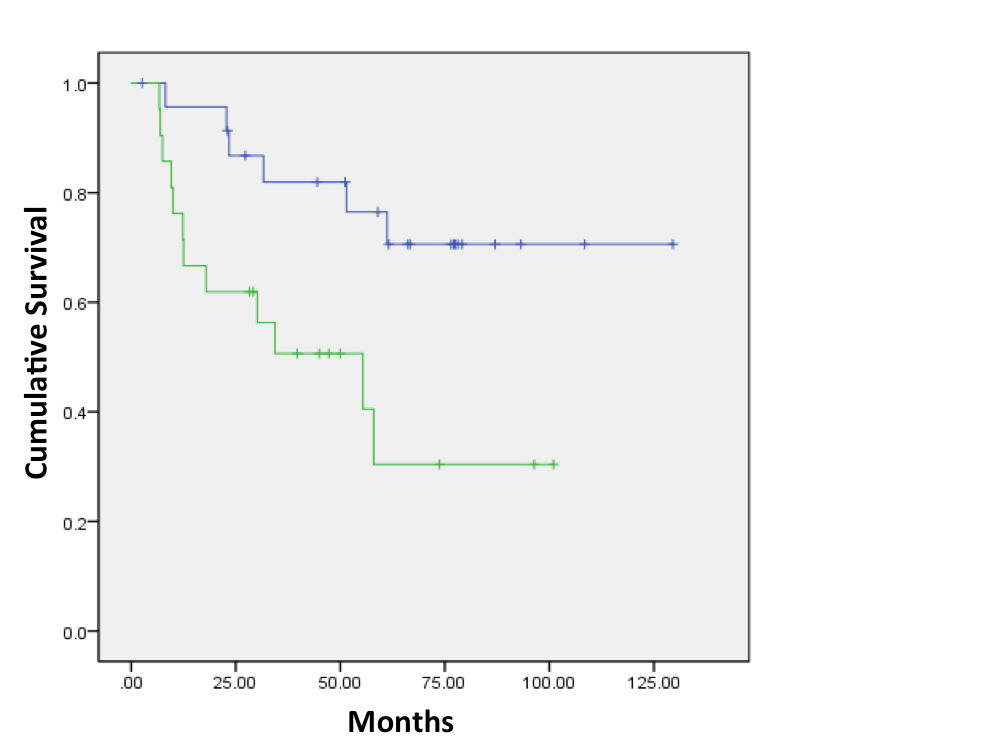

Supplement: Figure S4 — Disease free survival curves based on QTA. Without filtration, lower mean (green line, median DFS 55.4 months vs. blue line, median DFS not reached) was significantly associated with shorter DFS (p = 0.009). (TIF) [file pone.0100244.s004.tif]

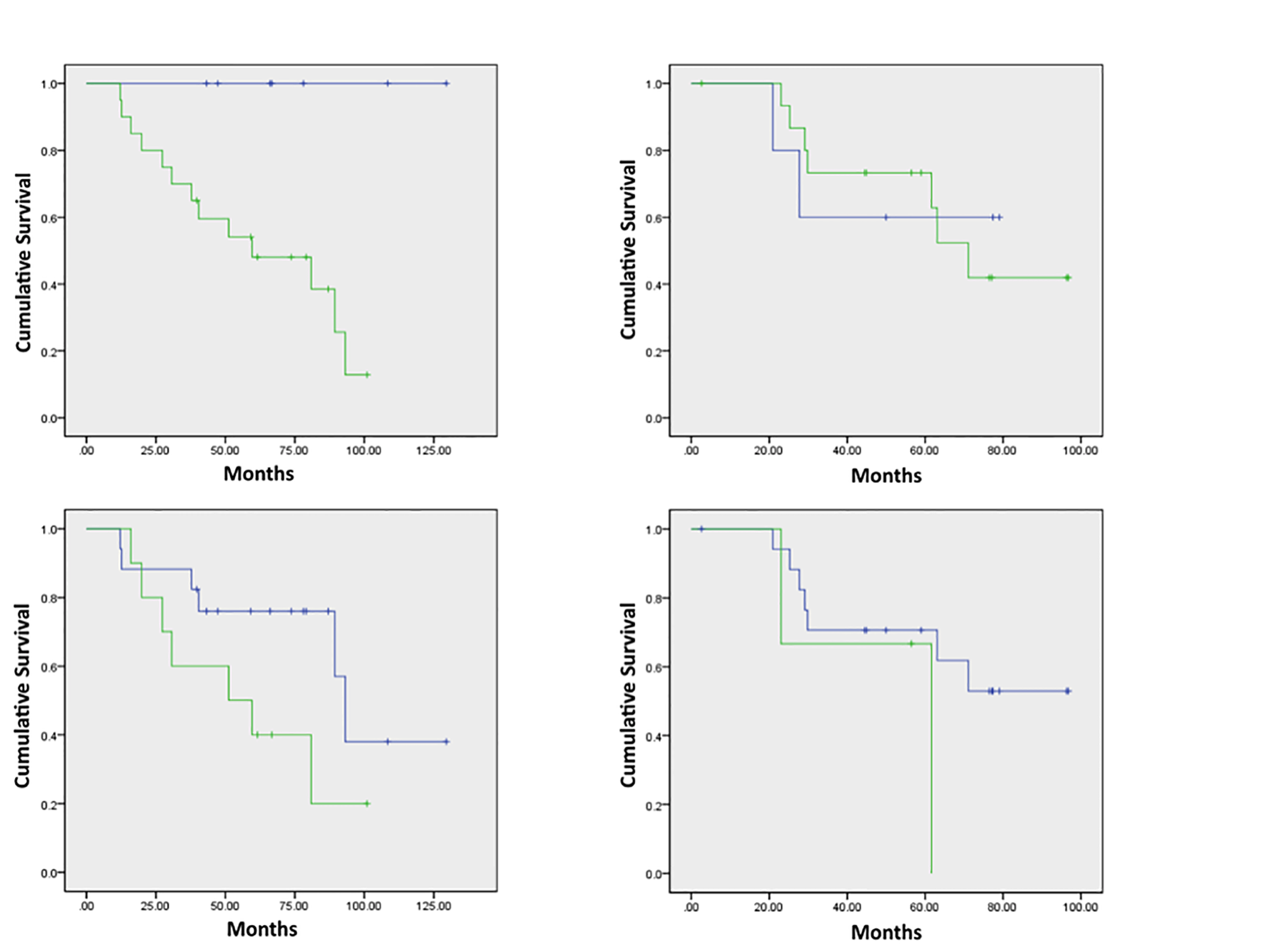

Supplement: Figure S5 — Overall survival curves based on QTA within K-ras mutant and pan-wildtype cases. With fine-texture, in patients with a K-ras mutant tumor, higher SD (blue line, median OS 59.6 months vs. green line, median OS not reached) was significantly associated with shorter OS (p = 0.038)(A, top left panel), while there were no significant differences in OS with SD in patients with pan-wildtype tumors (higher SD, blue line, median OS 71.1 months vs. lower SD, green line, median OS not reached)(B, top right panel). With coarse-texture, in patients with a K-ras mutant tumor, higher kurtosis (blue line, median OS 51.2 months vs. green line, median OS 93.2 months) was significantly associated with shorter OS (p = 0.044)(C, bottom left panel), while there were no significant differences in OS with kurtosis in patients with pan-wildtype tumors (higher kurtosis, blue line, median OS 61.6 months vs. lower kurtosis, green line, median OS not reached)(D, bottom right panel). (TIF) [file pone.0100244.s005.tif]

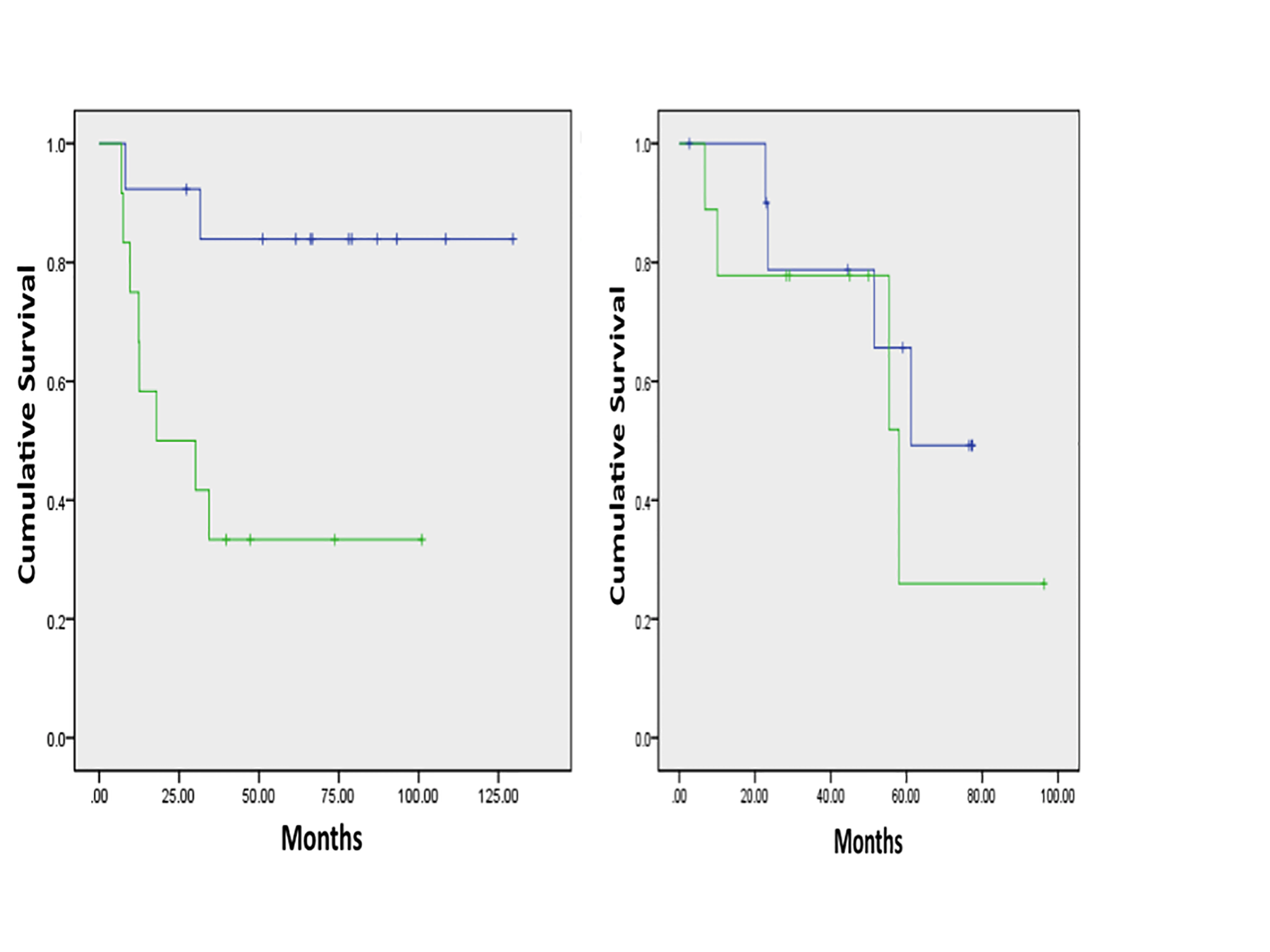

Supplement: Figure S6 — Disease free survival curves based on QTA within K-ras mutant and pan-wildtype cases. Without filtration in patients with a K-ras mutant tumor, lower mean (green line, median DFS 17.9 months vs. blue line, median DFS not reached) was significantly associated with shorter DFS (p = 0.015)(A, left panel). Without filtration in patients with a pan-wildtype tumor, there was no significant difference in DFS (green line, median DFS 61.2 months vs. blue line, median DFS 58.0 months)(B, right panel). (TIF) [file pone.0100244.s006.tif]
