# Supplementary material for: Noninvasive Image Texture Analysis Differentiates K-ras Mutation from Pan-Wildtype NSCLC and Is Prognostic
Source: PLoS One. 2014 Jul 2;9(7):e100244. doi: 10.1371/journal.pone.0100244 (PMC4079229; doi:10.1371/journal.pone.0100244)
Supplement: Table S1 — Clinical characteristics comparing pan-wildtype and K-ras mutant. (DOCX) [file pone.0100244.s007.docx]

**Supplementary Section**

**Table S1. Clinical characteristics comparing pan-wildtype and K-ras mutant**

| **Clinical Characteristic** | **P-value** |
| --- | --- |
| Age | 0.048 |
| Gender | 0.854 |
| Initial stage | 0.965 |
| Histology | 0.452 |
| Smoker | 0.245 |
| Adjuvant therapy | 0.587 |
| Relapse | 1.000 |
| Brain Metastasis | 0.267 |
| Vital status | 0.718 |
| DFS | 0.553 |
| OS | 0.486 |

Age is the only clinical characteristic with a significant difference between the 2 groups
